# Weight Bias Internalization Scale: Psychometric Properties and Population Norms

**DOI:** 10.1371/journal.pone.0086303

**Published:** 2014-01-29

**Authors:** Anja Hilbert, Sabrina Baldofski, Markus Zenger, Bernd Löwe, Anette Kersting, Elmar Braehler

**Affiliations:** 1 Integrated Research and Treatment Center AdiposityDiseases, University of Leipzig Medical Center, Leipzig, Germany; 2 Department of Medical Psychology and Medical Sociology, University of Leipzig Medical Center, Leipzig, Germany; 3 Department of Psychosomatic Medicine and Psychotherapy, University Medical Center Hamburg-Eppendorf, Hamburg, Germany; 4 Clinic of Psychosomatic Medicine and Psychotherapy, University of Leipzig Medical Center, Leipzig, Germany; 5 Clinic for Psychosomatics and Psychotherapy, University Clinic Giessen/Marburg, Giessen, Germany; University of Udine, Italy

## Abstract

**Objective:**

Internalizing the pervasive weight bias commonly directed towards individuals with overweight and obesity, co-occurs with increased psychopathology and impaired quality of life. This study sought to establish population norms and psychometric properties of the most widely used self-report questionnaire, the Weight Bias Internalization Scale (WBIS), in a representative community sample.

**Design and Methods:**

In a survey of the German population, N = 1158 individuals with overweight and obesity were assessed with the WBIS and self-report measures for convergent validation.

**Results:**

Item analysis revealed favorable item-total correlation of all but one WBIS item. With this item removed, item homogeneity and internal consistency were excellent. The one-factor structure of the WBIS was confirmed using confirmatory factor analysis. Convergent validity was shown through significant associations with measures of depressive and somatoform symptoms. The WBIS contributed to the explanation of variance in depressive and somatoform symptoms over and above body mass index. Higher WBIS scores were found in women than in men, in individuals with obesity than in individuals with overweight, and in those with lower education or income than those with higher education or income. Sex-specific norms were provided.

**Conclusions:**

The results showed good psychometric properties of the WBIS after removal of one item. Future research is warranted on further indicators of reliability and validity, for example, retest reliability, sensitivity to change, and prognostic validity.

## Introduction

Weight bias includes pervasive negative stereotypes and prejudice regarding an individual's overweight, such as attributions of responsibility or incompetence, and can extend to actual discrimination in multiple domains of life [1], [2]. Stigmatized individuals with overweight and obesity often have the tendency to internalize this weight bias, leading to feelings of incompetence, self-hate, or devaluation. Consequently, weight bias internalization has significant associations with depressive symptoms, anxiety, lower self-esteem, eating disorder psychopathology, social and behavioral problems, lower quality of life and health status, and greater health care utilization [3]–[8]. Weight bias internalization has been shown to have greater explanatory power of psychopathology over and above stigmatizing attitudes, experiences of discrimination, and body mass index (BMI, kg/m^2^) [9]–[12].

Despite the psychopathological relevance of weight bias internalization, only two self-report questionnaires are available for assessment [5], [9]. The most commonly used instrument, the Weight Bias Internalization Scale (WBIS) [9], measures the degree to which a respondent believes that negative stereotypes and self-statements about persons with overweight and obesity apply to herself or himself (11 items, e.g., “I hate myself for being overweight;” 1 = *strongly disagree* to 7 = *strongly agree*). Psychometric analyses in adult samples documented good internal consistency of the total mean score (.71≤Cronbach's α≤.94), corrected item-total correlations in the middle to upper range, a unidimensional factor structure, and convergent validity in the explanation of psychopathology as described above [8]–[12]. A re-analysis of the measure in adolescents seeking surgical treatment for obesity suggested the elimination of one item to yield unidimensionality, and Cronbach's α and corrected item-total correlations were slightly enhanced [4]. Overall, the WBIS was developed and evaluated in non-representative Internet-based community samples [9], [10], [12] and in smaller-sized clinical samples (n<200) [3], [4], [8]. Thus, establishing population norms and additional psychometric properties (including factorial, convergent, and discriminant validity, and item statistics) in a representative sample are needed. This study addressed these aspects for the German version of the WBIS.

## Materials and Methods

### Recruitment and Sample

In June and July 2012, a representative sample of the German population was recruited, with assistance by an independent agency specializing in market, opinion, and social research (USUMA; Berlin, Germany). In a three-stage random sampling procedure, sample point regions were selected from 320 regions, based on representative data; target households within these sample point regions were determined using a random route procedure; and target persons within these target households were selected using a kish selection grid. Inclusion criteria were age ≥14 years and fluent German.

Following this procedure, 4436 target households were randomly selected from all German states. Of these, *N* = 2515 individuals participated in the assessment, corresponding to a response rate of 56.7% (573 [12.9%] households could not be reached; 609 [13.7%] households refused to participate; 127 [2.9%] target persons could not be reached; 23 [0.5%] target persons were incapacitated; and 589 [13.3%] target persons refused to participate). Of the *N* = 2515 assessments, 5 (0.1%) were excluded from the analyses due to insufficient data. Thus, the sample consisted of *N* = 2510 assessments.

All participants were visited in-person. A maximum of four attempts were made to contact a target person. Participants were informed about the study by a trained research assistant, and told the purpose of the study was to investigate health and general behavior. Participants provided their oral informed consent prior to assessment (for minor participants, oral informed consent was also obtained from one parent). Oral consent is common in survey research in Germany. The ethical guidelines of the International Code of Marketing and Social Research Practise by the International Chamber of Commerce and the European Society for Opinion and Marketing Research were followed. The Ethics Committee of the University of Leipzig approved the methodological concept for the conduct of this study including the consent procedure according to which participants were included and assessed only if they had given their verbal consent (Approval No. 092-12-05032012). During the self-report assessment, the research assistant was present in order to assist with completion if he or she was asked for help. In accordance with this procedure, social desirability effects were limited, but cannot be excluded.

Body mass index (BMI, kg/m^2^) was calculated from self-reported weight and height. As the WBIS was originally designed to measure weight bias internalization, only overweight and obese participants with BMI≥25.0 kg/m^2^ were selected for the analyses (*N* = 1164 [46.9%]). Additionally, participants with incomplete data on the WBIS (i.e., one or more missing items) were excluded from the analyses. Thus, *N* = 1092 participants were retained for the final study sample.

Sample characteristics are shown in Table 1. The final study sample consisted of 514 (47.1%) women and 578 (52.9%) men between the ages of 14 and 89 (*M* = 53.90 years, *SD* = 16.12). Mean BMI was 28.30 kg/m^2^ (*SD* = 3.73, range 24.97–66.92 kg/m^2^); 870 individuals (79.7%) were overweight (BMI 25.0–29.9 kg/m^2^), and 222 (20.3%) were obese (BMI≥30.0 kg/m^2^). Less than 12 years of education were reported by 85.8%, 7.9% had a household income of <EUR 1000, 57.9% were married, 76.2% lived in the western part of Germany, and 98.4% had a German nationality.

**Table 1:** Sociodemographic characteristics (*N* = 1,092).

		Women (*N* = 514)	Men (*N* = 578)
		*N* (%)	*N* (%)
Age (years)	≤24	25 (4.9)	32 (5.5)
	25–34	58 (11.3)	51 (8.8)
	35–44	61 (11.8)	75 (13.0)
	45–54	87 (16.9)	113 (19.6)
	55–64	133 (25.9)	133 (23.0)
	65–74	99 (19.3)	142 (24.6)
	≥75	51 (9.9)	32 (5.5)
Weight status	Overweight (25.0–29.9 kg/m^2^)	392 (76.3)	478 (82.7)
	Obese (≥30.0 kg/m^2^)	122 (23.7)	100 (17.3)
Education (years)	<12	464 (90.3)	473 (81.8)
	≥12	50 (9.7)	105 (18.2)
Household income (EUR/month)	<1000	51 (10.0)	34 (6.0)
	≥1000	458 (90.0)	534 (94.0)
Marital status	Married	269 (52.3)	363 (62.8)
	Single, divorced, widowed	245 (47.7)	215 (37.2)
Residence	Eastern part of Germany	119 (23.2)	141 (24.4)
	Western part of Germany	395 (76.8)	437 (75.6)
Nationality	German	509 (99.0)	566 (97.9)
	Other	5 (1.0)	12 (2.1)

*Notes.* Calculation of % from valid cases (*N*).

### Measures

#### Weight Bias Internalization Scale (WBIS)

The English version of the WBIS was translated into German and controlled by a back-translation procedure through a licensed translator (for psychometric properties, see Introduction).

#### Beck Depression Inventory for Primary Care (BDI-PC)

The BDI-PC [13] is a widely used screening questionnaire for major depression that consists of seven items (e.g., symptoms of sadness, pessimism, and loss of pleasure). The items are rated on four-point scales, each of which consists of four different statements that reflect varying degrees of depressive symptom severity. A total sum score is computed with higher scores indicating greater severity of depressive symptoms. The scale shows good internal consistency (Cronbach's α = .86) and convergent validity. It was hypothesized that WBIS scores would be positively correlated with greater symptom severity.

#### Somatic Symptom Scale – 8 (SSS-8)

The SSS-8 [14] is the short form of the PHQ-15 [15] and was used to assess the severity of somatic symptoms such as stomachaches or headaches. The eight items are scored from 1 = *not bothered at all* to 5 = *bothered very strongly*, and a total sum score is computed, with higher scores indicating greater somatic symptom severity. The SSS-8 shows good internal consistency (Cronbach's α = .81) and convergent validity. A positive correlation between WBIS scores and greater severity of somatic symptoms was expected.

### Data Analytic Plan

#### Primary analyses

For psychometric analyses, item distributions were tested for normality using the Shapiro-Wilks normality test. Pearson's *r* was calculated for corrected item-total-correlations, and average inter-item correlation. Item difficulty was estimated as *p_m_* = sum of item scores/(*N* * maximal item score). Cronbach's α was computed as a measure of internal consistency. All psychometric analyses were performed for men and women separately.

#### Secondary analyses

Distributions of WBIS mean scores were analyzed using univariate General Linear Model analyses including Sex×Age×Weight status, with an additional inclusion of the factors Education, Household income, Marital status, or Nationality, respectively, in separate steps (for categories of sociodemographic variables see Table 1). Univariate and post-hoc test results were only interpreted when significant higher-order effects were found. To estimate effect sizes, partial η^2^ was reported when appropriate and interpreted according to Cohen [16] (η^2^: small: .01, medium: .06, large: .14). Based on the results of the Sex×Age×Weight status analysis, percentiles were determined for the total mean score of the WBIS.

Confirmatory factor analyses (CFAs), using maximum likelihood estimation, were conducted to evaluate the hypothesized one-factorial structure of the WBIS [9]. First, a CFA was conducted for the final sample with BMI≥25.0 kg/m^2^. The data were examined for normality using the Mardia test. In the case of multivariate non-normality, the Bollen-Stine bootstrap method was utilized [17]. The adequacy of fit was assessed using the following statistics: χ^2^ test, Tucker-Lewis Index (TLI), Comparative Fit Index (CFI), Root Mean Square Error of Approximation (RMSEA), and Standardized Root Mean Residual (SRMR). Goodness-of-fit indices were interpreted according to Hu and Bentler and Schermelleh-Engel et al. [18], [19].

To test the WBIS's convergent validity, Spearman's rank correlation coefficients were calculated between WBIS scores and measures for convergent validation. Hierarchical multiple regression analyses were run to assess impact of sex, age, BMI (block 1), and WBIS mean score (block 2) on depression and severity of somatic symptoms, respectively. In an additional step, an interaction term between sex * WBIS scores was included in both regression analyses. Effect size of prediction was evaluated according to Cohen [16] (R^2^: small: .01, medium: .09, large: .25).

A two-tailed α<.05 was applied for all statistical tests. Statistical analyses were performed using IBM SPSS Statistics and AMOS version 20.0. Data are available upon request.

## Results

### Primary Analyses

#### Item analysis

The percentage of missing item responses was low (*M* = 4.83%, *SD* = 0.50). Item distributions deviated significantly from normality (all *p*<.01). All items were positively skewed, except item 4 (“I wish I could drastically change my weight”), which was negatively skewed for women (Table 2). Most items had a low kurtosis. The difficulty indices were of medium size (.27≤*p_m_*≤.53). Corrected item-total correlations were in the middle to upper range (.47≤*r_it_*≤.78), except for item 1 (“As an overweight person, I feel that I am just as competent as anyone,” reverse scored). This item showed a negative correlation (*r_it_* = −.04) and was therefore removed from the WBIS, in accordance with Roberto et al. [4]. Thus, 10 items were retained as the final scale and used in all secondary analyses. Item homogeneity was optimal (mean inter-item correlation: *r* = .50; for the original 11 item scale: *r* = .40). Item statistics of the final scale are displayed in Table 2.

**Table 2:** Item characteristics of the Weight Bias Internalization Scale (*N* = 1,092).

Item or Variable	Women (*N* = 514)							Men (*N* = 578)						
	*M*	*SD*	Skewness	Kurtosis	*p_m_*	*r_it-o_*	*r_it-f_*	*M*	*SD*	Skewness	Kurtosis	*p_m-o_*	*r_it-o_*	*r_it-f_*
1. Feeling competent *(r)*	3.23	2.00	0.61	−0.78	0.46	−.02	–	3.34	2.09	0.53	−0.97	0.48	−.05	–
2. Less attractive	3.18	1.73	0.21	−1.04	0.45	.64	.67	2.70	1.69	0.60	−0.78	0.39	.68	.72
3. Anxious about being overweight	2.84	1.68	0.44	−0.98	0.41	.76	.77	2.37	1.53	0.83	−0.36	0.34	.77	.79
4. Wish to change weight	4.07	1.84	−0.21	−0.92	0.58	.53	.60	3.38	1.88	0.24	−1.04	0.48	.49	.56
5. Feeling depressed	2.95	1.80	0.52	−0.77	0.42	.76	.78	2.37	1.58	0.89	−0.25	0.34	.80	.81
6. Hate myself	2.40	1.61	0.91	−0.18	0.34	.76	.77	1.96	1.36	1.32	0.90	0.28	.77	.78
7. Judge value as a person	3.16	1.76	0.33	−0.87	0.45	.62	.62	2.59	1.63	0.64	−0.73	0.37	.63	.63
8. Deserving no fulfilling social life	1.99	1.36	1.25	0.81	0.28	.52	.48	1.81	1.27	1.49	1.34	0.26	.63	.61
9. Being OK *(r)*	3.33	1.74	0.39	−0.66	0.48	.49	.48	2.83	1.71	0.76	−0.22	0.40	.42	.39
10. Not feeling like true self	2.32	1.41	0.62	−0.93	0.33	.68	.67	1.99	1.36	1.22	0.48	0.28	.72	.72
11. Not being dated	2.72	1.60	0.54	−0.59	0.39	.69	.69	2.36	1.54	0.91	−0.08	0.34	.66	.68
WBIS mean score – original scale	2.93	1.11	0.49	−0.38				2.52	1.05	0.64	−0.31			
WBIS mean score – final scale	2.90	1.20	0.39	−0.51				2.44	1.14	0.68	−0.40			

*Notes. (r)*, reverse scored; *M*, mean; *SD*, standard deviation; *p_m_*, item difficulty; *r_it-o_*, *r_it-f_*, corrected item-total correlations for original 11 item and final 10 item scale of the Weight Bias Internalization Scale.

#### Reliability

The WBIS mean score showed excellent internal consistency (Cronbach's α = .91; for the original 11 item scale Cronbach's α = .87). Overall, excluding item 1 led to an improvement of item homogeneity and reliability.

### Secondary Analyses

#### Distributions of WBIS mean scores

A univariate analysis of WBIS mean scores by sex, age, and weight status showed significant main effects for sex and weight status with small effect sizes [sex: F(1, 1064) = 15.55, *p*<.01, η^2^ = .01; weight status: F(1, 1064) = 25.63, *p*<.01, η^2^ = .02], and a significant Sex×Age interaction with a small effect size [F(6, 1064) = 2.42, *p*<.05, η^2^ = .01]. Age (*p* = .36) and interactions of Sex×Weight status (*p* = .83), Age×Weight status (*p* = .68), and Sex×Age×Weight status (*p* = .64) yielded no significant results. Women had significantly higher WBIS scores than men (*p*<.01), and participants with obesity showed significantly higher scores than participants with overweight (obesity: *M* = 3.07, *SD* = 1.29; overweight: *M* = 2.55, *SD* = 1.15; *p<*.01). Post-hoc univariate General Linear Model analyses for the significant Sex×Age interaction in women resulted in higher WBIS scores at age 35–44 than at age 55–64 and 65–74 (35–44 years: *M* = 3.42, *SD* = 1.16; 55–64 years: *M* = 2.77, *SD* = 1.12; 65–74 years: *M* = 2.68, *SD* = 1.06; all *p*<.01), but yielded no differences between age groups in men (*p*>.05). Based on these findings, sex-specific percentile ranks were calculated and displayed in Table 3.

**Table 3:** Sex-specific norms of the Weight Bias Internalization Scale (*N* = 1,092).

Percentiles	Women (*N* = 514)	Men (*N* = 578)
1	1.00	1.00
5	1.10	1.00
10	1.40	1.10
15	1.60	1.20
20	1.70	1.40
25	1.90	1.50
30	2.05	1.60
35	2.30	1.70
40	2.40	1.80
45	2.60	2.00
50	2.80	2.20
55	2.90	2.30
60	3.20	2.50
65	3.40	2.80
70	3.70	3.10
75	3.90	3.30
80	4.00	3.60
85	4.10	3.90
90	4.45	4.00
95	5.03	4.50
99	5.90	5.40

In further univariate analyses, additional inclusion of single sociodemographic variables showed significant main effects for education and income with small effect sizes [education: F(1, 1038) = 4.32, *p*<.05, η^2^ = .00; income: F(1, 1028) = 7.39, *p*<.01, η^2^ = .01]. Marital status (*p* = .62) and nationality (*p* = .08) yielded no significant main effects. WBIS scores were significantly higher for participants with lower education than for participants with higher education (<12 years: *M* = 2.68, *SD* = 1.18; ≥12 years: *M* = 2.47, *SD* = 1.25; *p*<.05), and for participants with lower household income than for participants with higher income (<EUR 1000: *M* = 2.94, *SD* = 1.32; ≥EUR 1000: *M* = 2.63, *SD* = 1.18).

#### Factorial validity

Results of the CFAs for a one-factor model are provided in Table 4. For the first CFA (*N* = 1092), the one-factor model did not provide a good fit to the data as indicated by the significant χ^2^ test statistic. However, the *χ^2^* statistic is sensitive to sample size, so it is unclear whether this statistical significance was because of poor model fit or large sample size. Regarding the indices of model fit, CFI and SRMR indicated a good model fit, while the other indices were slightly lower or higher than recommended, but still in an acceptable range (17). Factor loadings were medium to high for all items (range .47–.85).

**Table 4:** Goodness-of-fit indices for a one-factor model of the Weight Bias Internalization Scale in two samples.

Sample	Bollen-Stine		TLI	CFI	RMSEA (90% CI)	SRMR
	χ^2^	*df*				
BMI≥25.0 kg/m^2^ (*N* = 1092)	502.94*	35	.90	.92	.11 (.10–.12)	.05
BMI≥25.0 kg/m^2^ and feeling overweight (*N* = 447)	183.96*	35	.91	.93	.10 (.08–.11)	.05

*Notes. df*, degrees of freedom; TLI, Tucker-Lewis Index; CFI, Comparative Fit Index; RMSEA, Root Mean Square Error of Approximation; CI, confidence interval; SRMR, Standardized Root Mean Residual; BMI, body mass index.

* *p*<.01.

#### Convergent validity

The WBIS showed significant positive correlations with the BDI-PC (*r* = 0.27; *p*<.01) and the SSS-8 (*r* = 0.24; *p*<.01).

#### Prediction of depression and somatic symptom severity

In order to determine whether and to what extent internalized weight bias predicted depression and somatic symptom severity, sex, age, BMI and WBIS mean scores were regressed on the BDI-PC and SSS-8 total scores, respectively. Regarding both depression and somatic symptom severity, sex, age and BMI explained a significant amount of variance in Step 1, while internalized weight bias significantly contributed to the explained variance in Step 2, both with small-to-medium effect sizes (see Tables 5, 6). Regarding gender differences in WBIS scores, in both regression analyses the additional inclusion of an interaction term between gender and WBIS scores yielded no further increase of explained variance (data available upon request).

**Table 5:** Prediction of depression by sex, age, and body mass index (Step 1) and Weight Bias Internalization Scale mean score (Step 2).

	*B*	SE	*β*
Step 1			
Sex	0.30	0.13	.07*
Age	0.02	0.00	.11**
BMI	0.09	0.02	.15**
Step 2			
Sex	0.09	0.13	.02
Age	0.02	0.00	.13**
BMI	0.06	0.02	.10**
WBIS mean score	0.50	0.06	.27**

*Notes*. *N* = 1092. *B*, unstandardized coefficient; *SE*, standard error; *β*, standardized coefficient; BMI, body mass index; WBIS, Weight Bias Internalization Scale.

* *p*<.05;

** *p*<.01.

*R^2^* = .04 for Step 1 (*p*<.01), *ΔR^2^* = .07 for Step 2 (*p*<.01).

**Table 6:** Prediction of somatic symptom severity by sex, age and body mass index (Step 1) and Weight Bias Internalization Scale mean score (Step 2).

	*B*	SE	*β*
Step 1			
Sex	0.84	0.23	.11**
Age	0.06	0.01	.23**
BMI	0.20	0.03	.18**
Step 2			
Sex	0.50	0.23	.06*
Age	0.06	0.01	.24**
BMI	0.15	0.03	.14**
WBIS mean score	0.78	0.10	.24**

*Notes*. *N* = 1092. *B*, unstandardized coefficient; *SE*, standard error; *β*, standardized coefficient; BMI, body mass index; WBIS, Weight Bias Internalization Scale.

* *p*<.05;

** *p*<.01.

*R^2^* = .10 for Step 1 (*p*<.01), *ΔR^2^* = .05 for Step 2 (*p*<.01).

## Discussion

This study provides the first comprehensive analysis of the WBIS psychometric properties in a representative population sample. The results confirmed good psychometric properties of the WBIS after removing one item and extended the evidence regarding item statistics, and discriminant and convergent validity. In addition, sex-specific population norms were provided, allowing rapid classifications of individual WBIS scores using population percentiles.

Item analysis showed that the WBIS leads to a low number of missing data (<5%). As the participants with overweight and obesity mostly endorsed item values indicating low to moderate weight bias internalization, all items deviated from normality and showed mostly flat distributions (low kurtosis) with a long tail to the right (positive skew). Relatedly, item difficulties were of medium size. Item-total correlations were favorable, with the exception of item 1 (“As an overweight person, I feel that I am just as competent as anyone;” reverse scored) that yielded an insufficient, negative score. Because of this negative score, item 1 was removed from the WBIS, consistent with Roberto et al.'s results in adolescents with obesity [4]. For the 10-item WBIS, corrected item-total correlations and item homogeneity were good and improved when compared to the 11-item WBIS. Internal consistency of the 10-item WBIS was excellent, which is consistent with previous literature [4].

Regarding validity, the CFA of the shortened WBIS yielded a one-factorial structure as postulated, and extended Roberto et al.'s results in adolescents with obesity [4] to adults with overweight and obesity. Regarding discriminant validity, weight bias internalization was greater in women than in men, and in women of middle age than in those of higher age, while for men, no age differences were found. These results are inconsistent with previous research in a small sample suggesting an absence of sex or age effects [4], but consistent with a current study using a modified version of the WBIS [12]. In addition, there is evidence that women are more often exposed to diverse forms of weight-related stigmatization and discrimination than men [20], [21] so that they may present with greater weight bias internalization. The difference between middle-aged versus higher-aged women could be related to increased obesity rates and pronounced body dissatisfaction in this lower age group, while at higher ages, body dissatisfaction tends to decrease [22], [23]. Weight bias internalization of young women who are at risk of a negative body image [23] may not have been higher than that of older women because of lower obesity rates at younger ages [24].

Further, individuals with obesity, identified on the basis of their self-reported weight and height, showed greater weight bias internalization than individuals with overweight. Previous research in small samples had mostly provided inconsistent associations with BMI [3], [9], [11], while two studies using another questionnaire or a modified WBIS version to assess weight bias internalization documented similar variations by weight status [5], [12]. As a novel aspect, we studied associations among weight bias internalization, and education and household income. Individuals with lower education or lower income showed greater weight bias internalization than those with higher education or higher income. A low socioeconomic position including low education has significant associations with obesity in Western industrialized countries [25], [26], and greater weight bias internalization is plausible in individuals with obesity who are pervasively exposed to weight bias. In contrast, income has previously shown more inconsistent associations with measures of obesity than education, presumably because income is less clearly associated with behaviors affecting energy balance (e.g., physical activity) [24]. Both low education and low income are also related to less positive self-beliefs [27], likely impacting greater weight bias internalization [7]. No further variation was found by marital status, residence, or nationality.

Regarding convergent validity, the WBIS was associated with greater depressive symptoms, as expected [4], [11]. In addition, for the first time the WBIS was shown to be associated with greater somatoform symptoms. Taken together with prior results demonstrating a link with a range of mental disturbances, weight bias internalization appears to be a common factor that may increase vulnerability to psychopathology in overweight and obesity. In addition, weight bias internalization independently contributed to depression and somatoform symptoms over and above BMI, in accordance with previous literature [8], [9]. Longitudinal studies are needed in order to clarify causal associations with psychopathology and weight management. Thus far, one small-scale prospective clinical study suggested that weight bias internalization does not predict weight loss [3], and may therefore not represent a barrier to successful weight management.

A strength of this study includes the large sample, drawn from a survey representative of the German population regarding age and sex [28]. Presumably because fluent German was an inclusion criterion, participants with another nationality than German were, however, underrepresented. More research is desirable on self-stigma in migrant groups. A further limitation is that the definition of overweight and obesity was based on self-reported height and weight, commonly leading to an underestimation of these aspects, and thus to an underestimation of prevalence rates of obesity [29]. Because of the self-reported nature of body weight and height, norms were not given for overweight and obesity separately. Of note, because the WBIS addresses weight bias in the overweight spectrum, persons of normal weight or underweight cannot answer most WBIS items and were therefore not included in this report. Future research should consider reformulating the WBIS items so that they apply to all weight groups, enabling comparisons among them [12].

Overall, this study established good psychometric properties of the shortened WBIS. The provision of norms is essential to identify individuals at increased risk of psychopathology and in need of interventions to reduce weight bias internalization [30], [31]. Future research is warranted on additional indicators of reliability and validity, for example, retest reliability, sensitivity to change, and prognostic validity.

## References

[1] Puhl RM, Heuer CA (2009) The stigma of obesity: a review and update. Obesity 17: 941–64. 19165161 10.1038/oby.2008.636

[2] Puhl RM, Latner JD (2007) Stigma, obesity, and the health of the nation's children. Psychol Bull 133: 557–80. 17592956 10.1037/0033-2909.133.4.557

[3] Carels RA, Wott CB, Young KM, Gumble A, Koball A, et al (2010) Implicit, explicit, and internalized weight bias and psychosocial maladjustment among treatment-seeking adults. Eat Behav 11: 180–5. 20434066 10.1016/j.eatbeh.2010.03.002

[4] Roberto CA, Sysko R, Bush J, Pearl R, Puhl RM, et al (2012) Clinical correlates of the Weight Bias Internalization Scale in a sample of obese adolescents seeking bariatric surgery. Obesity 20: 533–9. 21593805 10.1038/oby.2011.123 PMC3481186

[5] Lillis J, Luoma JB, Levin ME, Hayes SC (2010) Measuring weight self-stigma: The Weight Self-stigma Questionnaire. Obesity 18: 971. 19834462 10.1038/oby.2009.353

[6] Puhl RM, Moss-Racusin CA, Schwartz MB (2007) Internalization of weight bias: Implications for binge eating and emotional well-being. Obesity 15: 19–23. 17228027 10.1038/oby.2007.521

[7] Hilbert A, Braehler E, Haeuser W, Zenger M (2013) Weight bias internalization, core self-evaluation, and health in overweight and obese persons. Obesity 2013 Jul 9. doi: 10.1002/oby.20561. [Epub ahead of print] 10.1002/oby.20561 23836723

[8] Latner JD, Durso LE, Mond JM (2013) Health and health-related quality of life among treatment-seeking overweight and obese adults: associations with internalized weight bias. J Eat Disord 1: 3. 24764526 10.1186/2050-2974-1-3 PMC3776203

[9] Durso LE, Latner JD (2008) Understanding self-directed stigma: Development of the Weight Bias Internalization Scale. Obesity 16: S80–S6. 18978768 10.1038/oby.2008.448

[10] Durso LE, Latner JD, Hayashi K (2012) Perceived discrimination is associated with binge eating in a community sample of non-overweight, overweight, and obese adults. Obes Facts 5: 869–80. 23258192 10.1159/000345931

[11] Durso LE, Latner JD, White MA, Masheb RM, Blomquist KK, et al (2012) Internalized weight bias in obese patients with binge eating disorder: Associations with eating disturbances and psychological functioning. Int J Eat Disord 45: 423–7. 21717488 10.1002/eat.20933 PMC3184343

[12] Pearl RL, Puhl RM (2013) Measuring internalized weight attitudes across body weight categories: Validation of the Modified Weight Bias Internalization Scale. Body Image 2013 Oct 4. doi: 10.1016/j.bodyim.2013.09.005. [Epub ahead of print] 10.1016/j.bodyim.2013.09.005 24100004

[13] Beck AT, Guth D, Steer RA, Ball R (1997) Screening for major depression disorders in medical inpatients with the Beck Depression Inventory for Primary Care. Behav Res Ther 35: 785–91. 9256522 10.1016/s0005-7967(97)00025-9

[14] Gierk B, Kohlmann S, Kroenke K, Spangenberg L, Zenger M, et al (In press) The Somatic Symptom Scale – 8: A Brief Measure of Somatic Symptom Burden. JAMA Intern Med 10.1001/jamainternmed.2013.12179 24276929

[15] Kroenke K, Spitzer RL, Williams JB (2002) The PHQ-15: validity of a new measure for evaluating the severity of somatic symptoms. Psychosom Med 64: 258–66. 11914441 10.1097/00006842-200203000-00008

[16] Cohen J (1988) Statistical power analysis for the behavioral sciences. 2nd ed. Hillsdale, NJ: Erlbaum. 567 p.

[17] Bollen KA, Stine RA (1992) Bootstrapping goodness-of-fit measures in structural equation models. Sociol Methods Res 21: 205–29.

[18] Hu LT, Bentler PM (1999) Cutoff criteria for fit indexes in covariance structure analysis: Conventional criteria versus new alternatives. Struct Equ Modeling 6: 1–55.

[19] Schermelleh-Engel K, Moosbrugger H, Müller H (2003) Evaluating the fit of structural equation models: Tests of significance and descriptive goodness-of-fit measures. MPR-online 8: 23–74.

[20] Hansson LM, Näslund E, Rasmussen F (2010) Perceived discrimination among men and women with normal weight and obesity. A population-based study from Sweden. Scand J Public Health 38: 587–96. 20529966 10.1177/1403494810372266

[21] Han E, Norton EC, Powell LM (2011) Direct and indirect effects of body weight on adult wages. Econ Hum Biol 9: 381–92. 21820369 10.1016/j.ehb.2011.07.002

[22] Mensink GBM, Schienkiewitz A, Haftenberger M, Lampert T, Ziese T, et al (2013) Übergewicht und Adipositas in Deutschland – Ergebnisse der Studie zur Gesundheit Erwachsener in Deutschland (DEGS1). Bundesgesundheitsblatt Gesundheitsforschung Gesundheitsschutz 56: 786–794. 23703499 10.1007/s00103-012-1656-3

[23] Hilbert A, de Zwaan M, Braehler E (2012) How frequent are eating disturbances in the population? Norms of the Eating Disorder Examination-Questionnaire. PLOS ONE 7: e29125. 22279527 10.1371/journal.pone.0029125 PMC3261137

[24] Kurth BM (2012) Erste Ergebnisse aus der “Studie zur Gesundheit Erwachsener in Deutschland” (DEGS). Bundesgesundheitsblatt Gesundheitsforschung Gesundheitsschutz 55: 980–90. 10.1007/s00103-014-2053-x 25267318

[25] El-Sayed AM, Scarborough P, Galea S (2012) Unevenly distributed: A systematic review of the health literature about socioeconomic inequalities in adult obesity in the United Kingdom. BMC Public Health 9: 12–8. 10.1186/1471-2458-12-18 PMC3293718 22230643

[26] Ball K, Crawford D (2005) Socioeconomic status and weight change in adults: a review. Soc Sci Med 60: 1987–2010. 15743649 10.1016/j.socscimed.2004.08.056

[27] Schöllgen I, Huxhold O, Schüz B, Tesch-Römer C (2011) Resources for health: differential effects of optimistic self-beliefs and social support according to socioeconomic status. Health Psychol 30: 326–35. 21553976 10.1037/a0022514

[28] DESTATIS (Federal Statistical Office) (2013) Population 2013. Available: https://www.destatis.de/DE/ZahlenFakten/GesellschaftStaat/Bevoelkerung/Bevoelkerung.html. Accessed 2013 June 27.

[29] Visscher TL, Viet AL, Kroesbergen IH, Seidell JC (2006) Underreporting of BMI in adults and its effect on obesity prevalence estimations in the period 1998 to 2001. Obesity 14: 2054–63. 17135623 10.1038/oby.2006.240

[30] Hilbert A, Tuschen-Caffier B (2010) Essanfaelle und Adipositas: Ein Manual zur Kognitiv-Behavioralen Therapie der “Binge-Eating”-Stoerung. Goettingen: Hogrefe. 118 p.

[31] Li W, Rukavina P (2009) A review on coping mechanisms against obesity bias in physical activity/education settings. Obes Rev 10: 87–95. 18828779 10.1111/j.1467-789X.2008.00528.x

